# *Tropheryma whipplei* as a Cause of Epidemic Fever, Senegal, 2010–2012

**DOI:** 10.3201/eid2207.150441

**Published:** 2016-07

**Authors:** Hubert Bassene, Oleg Mediannikov, Cristina Socolovschi, Pavel Ratmanov, Alpha K. Keita, Cheikh Sokhna, Didier Raoult, Florence Fenollar

**Affiliations:** Aix-Marseille Université, Marseille, France and Dakar, Senegal (H. Bassene, O. Mediannikov, C. Socolovschi, P. Ratmanov, A.K. Keita, C. Sokhna, D. Raoult, F. Fenollar);; Far Eastern State Medical University, Khabarovsk, Russia (P. Ratmanov)

**Keywords:** *Tropheryma whipplei*, fever, epidemic fever, bacteremia, Whipple disease, bacteria, Senegal

## Abstract

The bacterium *Tropheryma whipplei*, which causes Whipple disease in humans, is commonly detected in the feces of persons in Africa. It is also associated with acute infections. We investigated the role of *T. whipplei* in febrile patients from 2 rural villages in Senegal. During June 2010–March 2012, we collected whole-blood finger-prick samples from 786 febrile and 385 healthy villagers. *T. whipplei* was detected in blood specimens from 36 (4.6%) of the 786 febrile patients and in 1 (0.25%) of the 385 apparently healthy persons. Of the 37 *T. whipplei* cases, 26 (70.2%) were detected in August 2010. Familial cases and a potential new genotype were observed. The patients’ symptoms were mainly headache (68.9%) and cough (36.1%). Our findings suggest that *T. whipplei* is a cause of epidemic fever in Senegal.

## Abstract 2

Findings suggest that the bacterium has role in febrile episodes, is contagious, and has an epidemic character.

Determining the etiologic causes of febrile illness in tropical settings provides public health and local community benefits. In the context of a decline in malaria cases in many parts of sub-Saharan Africa, the few studies that have been conducted in recent years to analyze the burden of bacterial infections used traditional blood cultures and identified typhoid fever and *Streptococcus pneumoniae* as the leading documented causes of nonmalarial bloodstream infections (*1*–*3*). However, this method does not enable the identification of intracellular organisms, and most causes of fever remain unknown. In 2008, we initiated a study of the etiologies of fevers of unknown origin in Africa, particularly in Senegal. Our preliminary studies showed the presence of previously known pathogenic microorganisms, such as *Borrelia crocidurae*, *Rickettsia felis*, *R. conorii*, and *Coxiella burnetii*, and the unexpected presence of *Tropheryma whipplei* (*4*–*9*).

*T. whipplei* was first considered to be an uncommon bacterium that causes Whipple disease, a rare chronic disease (*10*). However, *T. whipplei* is in fact a common bacterium associated with various conditions, such as acute infections (pneumonia and gastroenteritis) and chronic infections (classic Whipple disease and other infections without digestive involvement, including endocarditis and encephalitis) (*10*–*19*). *T. whipplei* can also be carried in human feces and, less commonly, in the saliva (*20*–*23*); carriage prevalence varies by the age and exposure of the population and by geographic area (*21*–*30*).

*T. whipplei* is highly prevalent in rural Senegal, where carriage rates reach 75% among children <2 years of age, and overall seroprevalence is 72% (*21*–*26*). In our preliminary study in Senegal, which was conducted in 2 villages (Dielmo and Ndiop) during December 2008–July 2009, we detected *T. whipplei* bacteremia in 6.4% of the analyzed specimens (*8*). Bacteremia was significantly associated with cough, but no link to feces carriage was observed (*8*). However, our study had several limitations, such as a small number of febrile patients, no local control group of afebrile persons, and a short study period. In this same area, we recently showed that humans comprise the only source of *T. whipplei* among the populations in whom the bacterium is highly prevalent. Moreover, our findings showed that limited access to toilets and exposure to human feces was associated with the high prevalence of *T. whipplei*, suggesting that these conditions may facilitate fecal–oral transmission of the bacterium (*31*). To better characterize *T. whipplei* bacteremia, we extended our analysis, beginning in 2010, in this same area of rural Senegal to include the collection of >1,000 blood samples from healthy persons and ambulatory patients with acute fever.

## Materials and Methods

We conducted the study during June 2010–March 2012 in Senegal’s rural Sine-Saloum area, a dry sahelian ecosystem with 2 typical seasons: dry (November–May) and rainy (June–October). We obtained written consent for every person included in the study. The National Ethics Committee of Senegal approved the study.

### Participants

Study participants included 786 febrile patients at the healthcare center for the villages of Dielmo and Ndiop; 78% of the patients were <15 years of age, and the sex ratio was 1:1. For all patients with fever (defined as axillary temperature of >37.5°C), we conducted a medical examination, completed a questionnaire, and collected a whole-blood finger-prick sample (200-μL [4 drops]) (*8*). In parallel, we collected blood samples from a control group of 385 healthy, afebrile villagers; 62.5% of these study participants were <15 years of age, and the sex ratio was 1:1.

### Molecular Analyses

#### DNA Extraction

For DNA extraction, we used a BioRobot EZ1 Workstation (QIAGEN, Courtaboeuf, France) according to the manufacturer’s instructions. Extraction was performed in Senegal, and specific quantitative real-time PCR (qPCR) was performed in France.

#### Specific qPCR

We used a 7900HT-thermocycler (Applied Biosystems, Foster City, CA, USA) with the QuantiTect-Probe PCR Kit (QIAGEN) to perform qPCR. First, we analyzed specimens for *T. whipplei* by using the primer pair Twhi3F (5′-TTG TGT ATT TGG TAT TAG ATG AAA CAG-3′)/Twhi3R (5′-CCC TAC AAT ATG AAA CAG CCT TTG-3′) and the specific Twhi3 probe (6-FAM-GGG ATA GAG CAG GAG GTG TCT GTC TGG-TAMRA). For specimens with positive results, we ran a second, confirmatory qPCR with the Twhi2F (5′-TGA GGA TGT ATC TGT GTA TGG GAC A-3′)/Twhi2R (5′-TCC TGT TAC AAG CAG TAC AAA ACA AA-3′) primer pair and the specific Twhi2 probe (6-FAM-GAG AGA TGG GGT GCA GGA CAG GG-TAMRA) (*8*,*21*). To validate the assays, we included positive (*T. whipplei*) and negative (PCR mix) controls in each run, as previously reported (*8*,*21*).

We considered samples to be *T. whipplei*–positive if qPCR results for the 2 specific genes were positive at a log-based fluorescence cycle threshold (C_t_) of <38. We used qPCR for the β-actin housekeeping gene, as previously described (*7*), to check the quality of DNA handling and blood specimen extraction; only positive samples were considered reliable.

### Genotyping

We performed genotyping of *T. whipplei* as previously described (*32*). We attempted to amplify and sequence each of 4 multispacer sequences (TW133, ProS, SecA, and Pro184) from positive specimens. When sequences were obtained, we compared them with those available in the GenBank database and our internal laboratory database to determine their corresponding genotype.

### Statistical Analyses

We performed statistical analyses by using Epi Info 6 software (http://www.cdc.gov/epiinfo/index.html); results with p<0.05 were considered statistically significant. The corrected χ^2^ test or the Fisher exact test was used where indicated.

## Results

### Prevalence of *T. whipplei* Bacteremia

A total of 786 febrile patients and 385 healthy controls were included in the study, among whom 36 (4.6%) and 1 (0.25%), respectively, were positive for *T. whipplei* DNA (p<0.00007). The positive control participant was a 13-year-old boy who had low concentrations of *T. whipplei* DNA (C_t_ of 36.85 and 37.99). The C_t_ for febrile patients ranged from 26.10 to 36.41 (mean ISD 33.40 ± 2.53).

### Age Distribution

The prevalence of *T. whipplei* bacteremia was 4% (3/75) for febrile patients <12 months of age, 4.8% (12/250) for those 1–3 years of age, 4.2% (5/119) for those 4–6 years of age, 5.4% (9/167) for those 7–15 years of age, 2.7% (2/75) for those 16–29 years of age, and 5.2% (5/97) for those >30 years of age. Age data were not available for 3 patients. No significant differences in age distribution were observed.

### Clinical Manifestations

Clinical data were available for 786 febrile patients (Table 1). The main symptoms in the 36 *T. whipplei*–positive febrile patients were headache (23 [68.9%]), cough (13 [36.1%]), rhinorrhea (8 [22.2%]), nausea (5 [13.9%]), vomiting (4 [11.1%]), and diarrhea (3 [8.3%]). No significant clinical differences were observed by C_t_ level.

**Table 1:** Clinical manifestations observed in 786 febrile *Tropheryma whipplei–*positive or –negative patients in 2 villages, Dielmo and Ndiop in the Sine-Saloum area of Senegal, June 2010–March 2012.

Clinical manifestation	*T. whipplei–*positive patients, no. (%), n = 36	*T. whipplei*–negative patients, no. (%), n = 750	p value by χ^2^ test
Headache	23 (68.9)	439 (58.5)	0.52
Arthralgia	0	19 (2.5)	0.46
Myalgia	0	53 (7.0)	0.07
Diarrhea	3 (8.3)	39 (5.2)	0.3
Vomiting	4 (11.1)	94 (12.5)	0.56
Nausea	5 (13.9)	100 (13.3)	0.53
Abdominal pain	1 (2.8)	21 (2.8)	0.68
Cough	13 (36.1)	274 (36.5)	0.95
Expectoration	2 (5.6)	42 (5.6)	0.67
Otalgia	1 (2.8)	28 (3.7)	0.61
Otorrhea	0	2 (0.3)	0.91
Rhinorrhea	8 (22.2)	229 (30.5)	0.28
Burning urination	1 (2.8)	33 (4.4)	0.53
Rash	0	10 (1.3)	0.62
Meningeal signs	2 (5.5)	25 (3.3)	0.35

### Seasonality

All 36 *T. whipplei* cases detected among the 786 febrile patients were in the 466 patients tested during the June–October rainy season; no cases were detected among the 320 febrile patients sampled during the November–May dry season (p = 0.0000001). Moreover, 33 (92%) of these 36 cases were diagnosed during the 2010 rainy season, and the other 3 were diagnosed during August 2011 (2 cases) and October 2011 (1 case) (Figure). The highest prevalence of *T. whipplei* bacteremia cases was detected during August, when 28 (30%) of 93 febrile patients were found to be positive (19 [28%] of 73 patients in Dielmo and 9 [45%] of 20 patients in Ndiop). In fact, the data were affected by the high prevalence of cases observed in August 2010, which seemed to be indicative of an outbreak.

**Figure:**
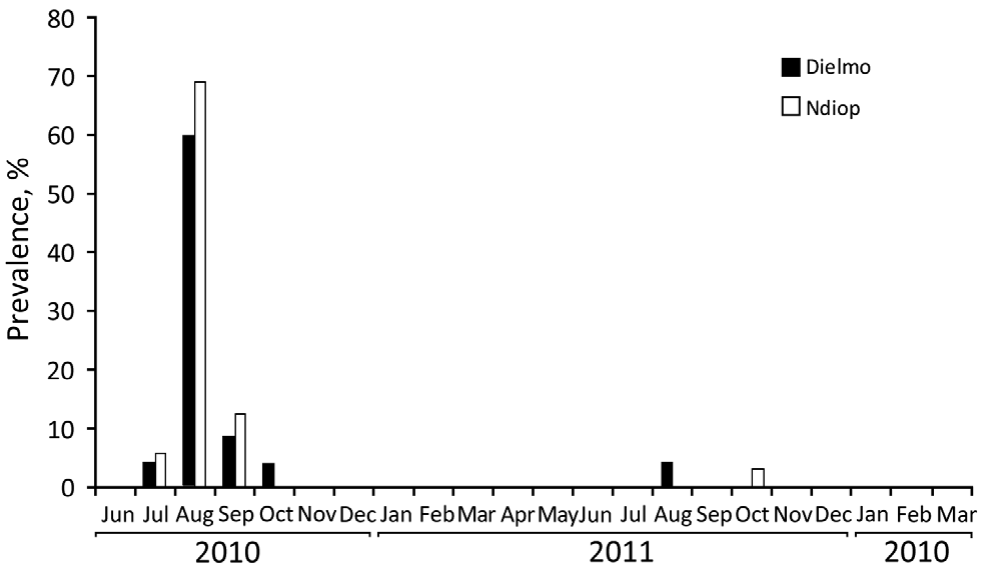
Monthly prevalence of *Tropheryma whipplei* bacteremia in Dielmo and Ndiop, Senegal, June 2010–March 2012. These 2 rural villages are located in the Sine-Saloum area, a dry sahelian ecosystem.

In July 2010, *T. whipplei* infection was detected in 2 febrile patients, an 18-year-old boy in Dielmo (case detected July 24) and a 15-year-old girl in Ndiop (case detected July 27). In August 2010, a total of 29 febrile patients from Dielmo were tested; 17 (58.5%) of the 29 patients had samples (18 total samples) positive for *T. whipplei* bacteremia. During the same month in Ndiop, 9 (69%) of 13 febrile patients had positive samples. In September 2010, 2 patients were positive in Dielmo and 1 in Ndiop, and in October, 2 patients were positive in Dielmo and none in Ndiop. For almost 1 year, all specimens from febrile patients were negative for *T. whipplei*. Then, in August 2011, only 2 patients were positive in Dielmo, and in October 2011, only 1 patient was positive in Ndiop.

### Treatment and Follow-Up

Data about antimicrobial drug therapy was available for 33 patients, 23 of whom benefited from treatment with amoxicillin (18 patients), metronidazole (3 patients), or cotrimoxazole (2 patients). In Dielmo, 24 specimens from 23 patients were positive for *T. whipplei*; 1 patient was sampled twice 15 days apart, and both specimens were positive. For 17 patients, blood specimens were also sampled during other febrile episodes. Nine specimens from 5 patients were sampled from 15 days to 13 months before the positive sample was detected, and 43 specimens from 17 patients were sampled from 3 weeks to 16 months after the positive sample was detected; all of these samples were negative. Moreover, our previously published data (*8*) included test results for a 4-year-old boy who was diagnosed with *T. whipplei* bacteremia in January 2009 (19 months before August 2010). Four other blood specimens from this patient were tested 1 month before (1 sample) or 4, 11, and 15 months after (3 samples) the positive specimen was detected, and all were negative for *T. whipplei*.

In Ndiop, 12 specimens from 12 patients were positive. For 8 of these patients, blood specimens were sampled during other febrile episodes. The specimen for 1 patient was sampled 1 month before the positive sample, and 9 specimens from 6 patients were sampled from 7 weeks to 18 months after the positive samples; all of these specimens were negative. No data were available for these patients about antibody response against *T. whipplei*.

### Genotyping

Because of the lack of specimens available for genotyping and the low sensitivity of genotyping, we could obtain multispacer sequences for only 8 patients at the time of the 2010 peak in *T. whipplei* bacteremia cases (Table 2). The *T. whipplei* genotype corresponds to the concatenation of the 4 spacers (TW133-ProS-SecA-Pro184); however, TW133 sequencing was not successful, so the corresponding spacer was not available (NA) for any of the patients. ProS sequence was obtained for 5 patients, SecA for 6 patients, and Pro184 for all patients. For 4 patients, 3 spacers were available, enabling the detection of the same multispacer sequence combination (NA-7-2-1) for the 4 patients. For another 4 patients, 2 spacers were available, enabling the detection of the NA-7-NA-1 combination for 2 of the patients and the NA-NA-2-1 combination for the other 2 patients. None of the potential combinations has previously been sequenced in Senegal. Moreover, the NA-7-2-1 combination has also not previously been detected in any other area of the world and is thus a new genotype. Overall, our data suggest that the same genotype was detected in Dielmo and Ndiop during the summer of 2010. However, *T. whipplei* genotyping was performed (sometimes only partially) for only 8 of 36 patients, so we can only suspect, but not confirm, that an epidemic clone was present and that an outbreak was ongoing at that time.

**Table 2:** *Tropheryma whipplei* multispacer typing results for 8 patients in the Sine-Saloum area of Senegal, 2010*

Patient no.	Age, y/sex	Sampling date	Village	Household no.	Spacers			
					TW133	ProS	SecA	Pro184
1	1/M	2010 Aug 4	Dielmo	14	NA	NA	2	1
2	1/M	2010 Aug 10	Dielmo	39	NA	7	2	1
3	5/M	2010 Aug 16	Dielmo	19	NA	NA	2	1
4	1/F	2010 Aug 22	Dielmo	6	NA	7	NA	1
5	4/M	2010 Aug 24	Dielmo	39	NA	7	2	1
6	13/F	2010 Jul 27	Ndiop	2	NA	7	2	1
7	2/F	2010 Aug 6	Ndiop	38	NA	7	2	1
8	2/M	2010 Aug 13	Ndiop	10	NA	7	NA	1

*NA, not available.

### Affected Households

In Dielmo during the peak of the August 2010 outbreak, multiple persons in several households were positive for *T. whipplei* bacteremia: 4 of 6 persons in household no. 19, 3 of 4 persons in household no. 39, 2 of 2 persons in household no. 9, and 2 of 3 persons in household no. 14. In Ndiop, 2 of 2 persons in household no. 3 and 2 of 3 persons in household no. 8 were positive for *T. whipplei* bacteremia. Of note, during this time, the family in household no. 39 had a furnace in which they baked bread that they marketed locally. In December 2010, most of the family left the village and the furnace was shut down; no additional *T. whipplei* bacteremia cases were subsequently observed.

## Discussion

We report the detection of *T. whipplei* DNA in the blood of patients in Dielmo and Ndiop, Senegal. The validity of our data is based on strict experimental procedures and controls, including rigorous positive and negative controls, used to validate test results. In addition, we confirmed each positive PCR result by the successful amplification of an additional specific DNA sequence, and we performed *T. whipplei* genotyping on several specimens. We also showed that the presence of *T. whipplei* in blood is significantly linked to the presence of fever; *T. whipplei* DNA was detected (at a low level) in the blood of only 1 afebrile person in the study area. Moreover, we included a control group of afebrile persons from the same area, thereby reinforcing the validity of our data. Indeed, several well-known pathogens have been detected in recently analyzed specimens from healthy persons. For example, *Plasmodium falciparum* has been detected in 32% of blood specimens from healthy, afebrile persons in Senegal (*33*); respiratory viruses, including influenza virus, have been detected in 12% of nasopharyngeal samples from symptom-free Hajj pilgrims (*34*); and *S. pneumoniae* has been detected in 6.3% of blood specimens from afebrile children in Tanzania (*35*). Thus, because of the significantly higher prevalence of *T. whipplei* among febrile patients compared with healthy controls, we suspect that this microorganism is a pathogenic agent.

The overall prevalence of *T. whipplei* bacteremia is 4.6%. However, in August 2010, we observed a peak in *T. whipplei* bacteremia cases in Dielmo and Ndiop, where *T. whipplei* was involved in more than half of the observed cases of fever. This peak corresponds to a short outbreak of *T. whipplei* bacteremia with 1 potential genotype. A similar new genotype was observed for the patients from Dielmo and Ndiop for whom genotyping was available at the time of the outbreak. To date, 35 different *T. whipplei* genotypes have been detected in Senegal, but only 1 common genotype has been detected in Dielmo and Ndiop, even though the villages are 5 km apart (*25*). All of the other genotypes detected in the Sine-Saloum area were specific to each village, including the 2 that were more prevalent: genotype 52 was detected in 54% of feces samples in Dielmo, and genotype 49 was detected in 28% of feces samples from Ndiop (*25*).

Several familial cases also occurred during this outbreak. The family in household no. 39 in Dielmo was 1 of the most affected families: 3 of 4 persons living in the home had fever and *T. whipplei* bacteremia. Genotyping was available for 2 of these patients, both of whom exhibited the same potential genotype. The family in household no. 39 was involved in the management of a traditional oven for preparing bread, which was thoroughly cooked and sold directly to other residents. Since the departure of the baker and his family, no other outbreaks have been observed, and the prevalence of *T. whipplei* bacteremia has dramatically decreased. Thus, this family may have contributed to spread of the outbreak on a daily basis in Dielmo and possibly on a weekly basis at traditional markets, which served as the main contact between villagers from Dielmo and Ndiop. Also of note, no toilet facilities were present in household no. 39, and a link between a lack of toilet facilities and the high detection of *T. whipplei*, mainly in feces, has previously been reported (*31*). Thus, we hypothesize that *T. whipplei* was transmitted to customers who bought bread contaminated with infectious feces (*31*). Overall, all of our data confirm human-to-human transmission of the bacterium (*22*,*2*3,*26*,*31*).

One of the main symptoms among febrile patients with *T. whipplei* bacteremia is cough (36.1%). In our preliminary study of *T. whipplei* bacteremia, cough was also the main manifestation observed (*36*). Thus, *T. whipplei* could be involved in respiratory infections (*13*,*14*,*36*,*37*). However, the presence of cough in ≈36% of febrile patients who were either *T. whipplei*–positive or –negative may also suggest that this symptom was poorly specific.

Of note, a 4-year-old patient had 2 febrile episodes associated with *T. whipplei* bacteremia 18 months apart (*8*); however, it was not possible to make a distinction between relapse and reinfection because genotyping was not available (*38*). Blood specimens from this patient that we tested for *T. whipplei* before and after the last febrile episode were negative, confirming that the infection was acute. Thus, these data suggest that some patients may have several febrile episodes linked to *T. whipplei.*

*T. whipplei* bacteremia cannot be diagnosed in tropical regions that lack the proper laboratory facilities or in industrialized countries that lack or do not routinely perform molecular biology–based diagnostics due to the specific training, expensive reagents, and excessive time required to perform such tests. Moreover, even recent studies that have looked for causes of nonmalarial fevers, including by performing molecular detection in blood for intracellular bacteria, such as *R. felis*, have not included the molecular detection of *T. whipplei* (*39*). Thus, it is currently difficult to estimate the prevalence of *T. whipplei* bacteremia. In conclusion, the results of our large-scale study clearly confirm the role of *T. whipplei* in febrile episodes as well as its contagiousness and epidemic character.
